# Technical classification of professional cycling stages using unsupervised learning: implications for performance variability

**DOI:** 10.3389/fspor.2025.1661456

**Published:** 2025-10-15

**Authors:** Igor Garcia-Atutxa, Ekaitz Dudagoitia Barrio, Francisca Villanueva-Flores

**Affiliations:** ^1^ Escuela Politécnica Superior, Universidad Católica de Murcia (UCAM), Murcia, Spain; ^2^ University of Murcia, Murcia, Spain; ^3^ Centro de Investigación en Ciencia Aplicada y Tecnología Avanzada, Instituto Politécnico Nacional, Atlacholoaya, Morelos, Mexico

**Keywords:** professional cycling, unsupervised learning, clustering, performance variability, external load

## Abstract

**Introduction:**

In professional cycling, the technical characteristics of race stages significantly influence group dynamics and performance variability among competitors. However, stage classifications have traditionally been subjective, lacking a robust empirical foundation. This study aimed to develop an objective, technical classification of professional cycling stages using unsupervised learning (KMeans) and analyze how these categories relate to collective performance variability, measured by the coefficient of variation (CV) of finish times.

**Methods:**

Technical data and official results from 439 international race stages conducted between 2017 and 2023 were analyzed. The technical variables included distance, total vertical gain, average relative elevation, and percentages of paved and unpaved surfaces.

**Results:**

Cluster validation via Bootstrap analysis demonstrated high stability (mean silhouette index = 0.62 ± 0.03), confirming six clearly distinct technical stage groups. Results indicated that stages characterized by higher relative elevation and greater proportions of unpaved surfaces exhibited higher performance variability (higher CV),whereas less technically demanding stages showed lower variability; relative elevation emerged as the strongest predictor of CV (*β* = 0.42, *p* < 0.001), followed by unpaved percentage (*β* = 0.23, *p* < 0.01), distance (*β* = 0.18, *p* < 0.05), and vertical gain (*β* = 0.11, *p* < 0.05). Across 2017–2023, a broadly downward pattern in CV was observed, although a pooled linear-trend test with cluster fixed effects did not reach statistical significance (*p* = 0.315).

**Discussion:**

The lack of physiological data and possible confounding from unmeasured stage and team factors (e.g., weather, stage order, team tactics) limit causal inference. This empirical typology provides a valuable quantitative tool to optimize competitive strategies, plan targeted training based on stage type, and prevent cumulative fatigue and performance-related injuries in high-performance cycling. Future research incorporating direct physiological data is recommended to further explore the relationship between external and internal load in professional cycling.

## 1 Introduction

Professional road cycling is an endurance sport marked by considerable technical and physiological complexity. Cycling stages exhibit substantial variation in factors such as total distance, accumulated elevation gain, average gradient, and terrain composition (1–3). These technical characteristics significantly shape the tactical strategies employed by teams and directly influence the physiological distribution and collective performance dynamics within the peloton (4).

Traditionally, cycling stages have been classified into broad categories such as “flat”, “mountainous”, or “time trial”. However, this conventional classification tends to be subjective and often lacks empirical precision, potentially overlooking relevant technical combinations observed in actual racing conditions (5, 6). Recent studies have highlighted that objective technical variables, such as accumulated elevation, relative elevation per kilometer, and surface composition, critically impact muscular fatigue, sustainable power output, and recovery between consecutive efforts in professional cycling (7, 8). These variables define the “external load”, a central concept in performance physiology that determines the intensity and specificity of physical demands during prolonged competitions (9–11).

Recently, researchers have paid attention to how these technical characteristics affect not only individual performance but also the cohesion and collective durability of group performance during stage races (12). This variability may increase the risk of fatigue-related injuries and highlight the need for better strategic adaptation (13, 14).

In parallel, recent advances in data science and machine learning algorithms have revolutionized methodologies for classifying and analyzing sports data. Clustering methods, in particular, enable empirical classification of sports events based on objective data patterns, offering more precise and reproducible typologies than traditional classifications (15, 16). Beyond cycling, unsupervised learning has been increasingly applied across endurance sports. For example, in running to identify technique-based subgroups and their relation to running economy (17), in collegiate cross-country cohorts using hierarchical clustering to profile mechanics and risk factors (18), and in swimming to partition inertial measurement unit (IMU)-derived functional data into skill-related patterns (19), thereby broadening the methodological context relevant to our approach. In cycling, the application of these methodologies remains limited, though initial studies have demonstrated their potential to generate empirically grounded typologies of cycling stages, thus facilitating improved strategic planning and more effective training load management (20).

Nevertheless, despite the practical relevance of the relationship between objective technical stage classifications and variability in collective performance, this connection has received limited empirical exploration in recent sports literature. The coefficient of variation (CV) of finish times emerges as a key indicator for assessing how specific technical features affect peloton performance homogeneity or dispersion (21, 22).

Therefore, the primary objective of this study is to develop an empirical and objective classification of professional cycling stages using unsupervised learning methods, and to evaluate how these technical categories relate to collective performance dispersion, measured through the CV of finish times. The central hypothesis of this research is that stages with higher technical demands (high elevation, mixed surfaces) are significantly associated with greater collective performance dispersion, reflecting increased physiological and tactical fatigue.

This study provides a robust quantitative framework useful for strategic and physiological planning in professional cycling, directly contributing to the optimization of specific training approaches, the prevention of cumulative fatigue-related injuries, and an improved understanding of how external technical loads influence the internal physiological dynamics of professional cyclists. Practically, this typology helps coaches, sports scientists, and teams tailor stage-type–specific training, pacing/fueling, and roster/equipment choices, while anticipating fatigue to minimize performance decrements and injury risk across multi-stage races.

## 2 Materials and methods

**Figure 1:**
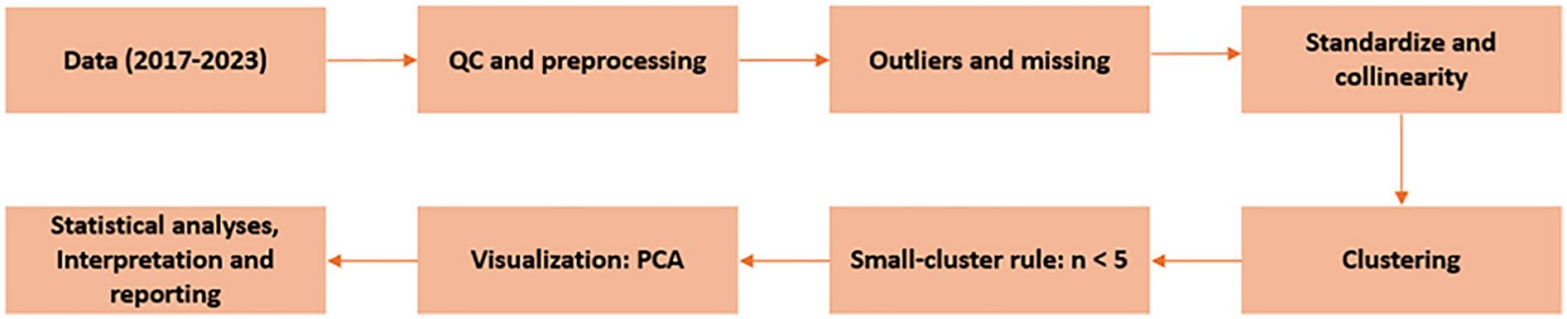
Methodological workflow. Data, QC/preprocessing, outliers/missing data, standardization/collinearity, clustering, small-cluster rule (*n* *<* 5; descriptive only), PCA, analysis and reporting.

Figure 1 summarizes the analysis pipeline: data (2017–2023); rule-based QC/preprocessing (completeness, duplicate/neutralized removal, plausibility bounds); outlier screening via the IQR rule with conservative handling of missing entries; z-score standardization (StandardScaler) and collinearity checks (VIF); K-means clustering with optimal k by silhouette and stability by bootstrap (1,000 resamples); small-cluster rule (*n* < 5, descriptive only); PCA visualization; and statistical analyses interpretation and reporting.

### 2.1 Study design

This retrospective study applied advanced statistical analysis and machine learning techniques to develop an objective and empirical classification of professional cycling stages. The dataset was derived from the publicly available “Geospatial Road Cycling Race Results Data Set” (23), which includes official race outcomes and technical details of stages from 2017 to 2023. The original data collection and validation procedures have been thoroughly described, ensuring analytical integrity and reliability for this study.

### 2.2 Data selection and analyzed variables

Data corresponding to 439 professional cycling stages from international races held between 2017 and 2023 were analyzed. Following the protocols and methodology described in (23), specific technical variables considered relevant according to recent literature were selected for analysis:

• Total distance (km): Official distance covered in each stage.
• Total vertical gain (m): Accumulated elevation gained throughout the stage.
• Average relative elevation (m/km): Average gradient calculated by dividing total elevation gain by total distance.
• Paved percentage (%): Proportion of the stage run on paved surfaces.
• Unpaved percentage (%): Proportion of the stage run on technical unpaved surfaces.
• Performance CV: Coefficient of variation (CV = SD/mean) of official finishing times across all classified riders (DNF/DSQ excluded), selected as a scale-invariant proxy that increases as the peloton fragments physiologically or tactically (e.g., breakaways, crosswinds, selective climbs), widening time gaps.

### 2.3 Statistical procedures and analytical techniques

All analyses were run in Python 3.10 (scikit-learn 1.4.2, pandas 2.2.2, NumPy 1.26.4, Matplotlib 3.9.2). Statistical significance was assessed at *α* = 0.05 (two-sided).

#### 2.3.1 Preprocessing

We applied a rule-based pipeline to ensure data quality and internal validity: retained stages with complete values for all modelling variables (Section 2.2) and an official finish time; removed duplicates; excluded neutralized or cancelled stages; and enforced plausibility bounds (e.g., strictly positive distance and time). Outliers were flagged using the interquartile-range rule (values <Q1 − 1.5 × IQR or >Q3 + 1.5 × IQR) and excluded if they violated pre-specified plausibility constraints or source metadata. Missingness was minimal: distance and finish times (for CV computation) were complete, except in cases of disqualification/withdrawal. Missing entries in total vertical gain and in road-surface composition (paved/unpaved %) were imputed as zero under a conservative assumption.

#### 2.3.2 Collinearity and scaling

Multicollinearity among technical predictors was examined via variance inflation factors (VIFs); all VIFs were below conventional thresholds, so no remedial action was required. Predictors were z-scored (StandardScaler) to equalize scales before analysis. Clusters with fewer than five stages were summarized descriptively and excluded from between-cluster inferential tests due to unreliable within-cluster variance (*n* ≤ 4) or its absence (*n* = 1).

#### 2.3.3 Clustering and validation

Stages were classified with K-means to obtain an objective technical typology (24). The number of clusters (k) was selected using the silhouette coefficient (range −1–1), computed with Euclidean distances on standardized features (25). Cluster stability was assessed by bootstrapping (1,000 resamples); the average silhouette across resamples exceeded 0.5, indicating stable separation.

#### 2.3.4 Visualization and complementary analyses

Principal component analysis (PCA) was used solely for low-dimensional visualization of cluster structure (26). Complementary analyses included: (i) ordinary least squares of annual mean CV on calendar year (2017–2023) to assess temporal trend; (ii) multiple linear regression of CV on standardized technical variables to quantify their partial associations; and (iii) descriptive comparisons of CV across clusters using boxplots and summary statistics (mean, SD).

## 3 Results

### 3.1 Technical classification of cycling stages through unsupervised clustering

**Figure 2:**
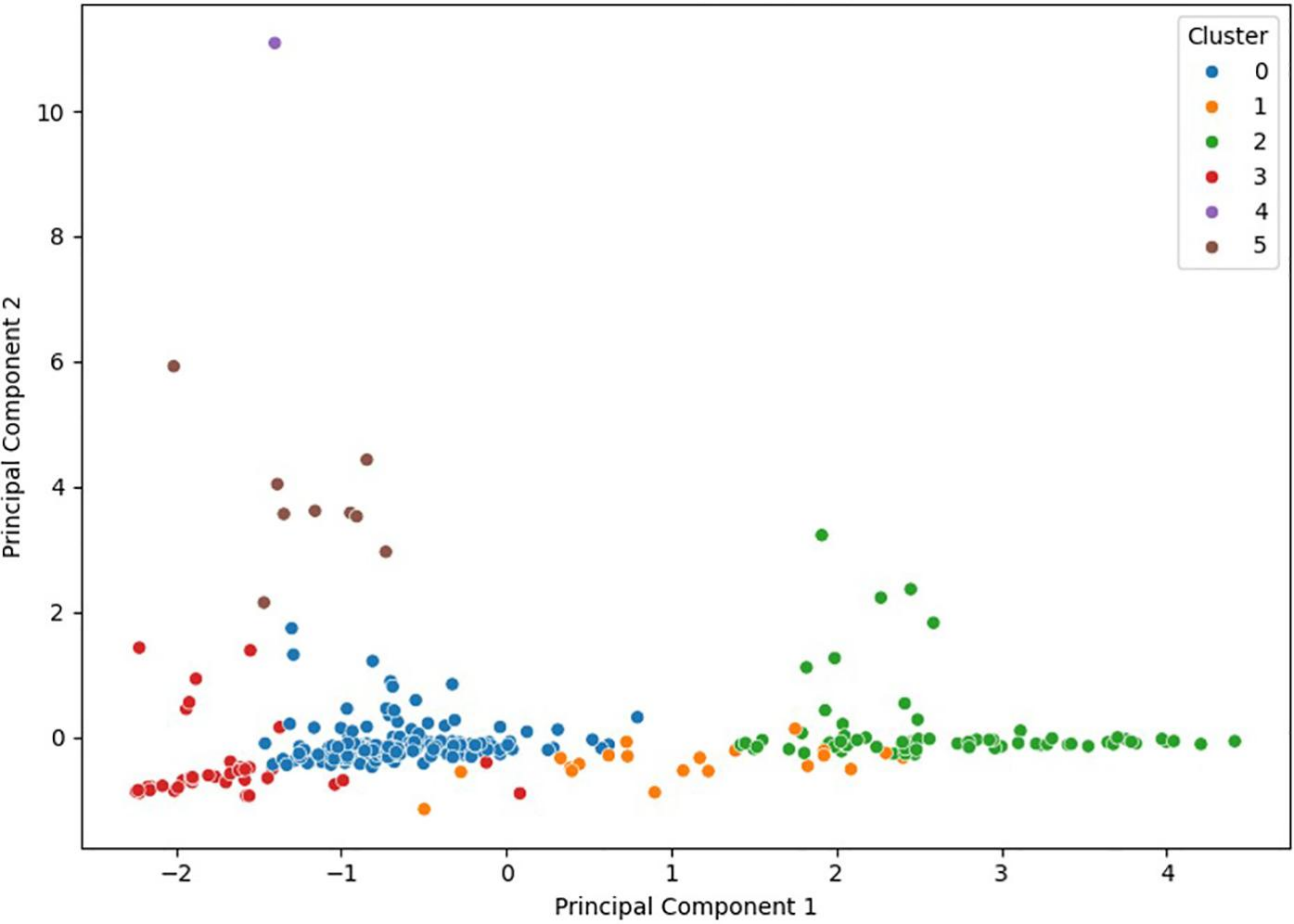
Two-dimensional PCA visualization illustrating the separation and distribution of the six technical clusters identified by the KMeans algorithm. Each point represents an individual cycling stage, with colors indicating the assigned technical cluster.

The application of the unsupervised KMeans clustering algorithm allowed the identification of six clearly distinct technical groups among the analyzed professional cycling stages. Visualization of these groups using PCA demonstrated clear separation, reflecting high internal coherence and strong external differentiation among the obtained clusters (Figure 2).

It is noteworthy that cluster 4 contains a single stage, indicating that this represents an exceptional and technically extreme case within the dataset. This single record is characterized by an especially high combination of relative elevation, distance, and a significant percentage of unpaved surface, clearly distinguishing it from the remaining clusters. Due to its uniqueness and limited statistical representation, this cluster will be excluded from subsequent comparative analyses to ensure the methodological validity and stability of the obtained results. Nevertheless, the practical and sporting relevance of this exceptional stage type suggests that future studies using larger datasets and incorporating additional analyses, including direct physiological variables, would be necessary to fully evaluate its implications for performance and strategic planning in professional cycling.

The five primary clusters were named according to their predominant technical characteristics to facilitate practical interpretation:

• Cluster 0 (Flat homogeneous stages): Flat profile with short distance, low relative elevation, and high proportion of paved surfaces.
• Cluster 1 (Medium-endurance stages): Stages of moderate to long distance, intermediate elevation, predominantly paved.
• Cluster 2 (Long mountainous stages): Stages with high distance, significant relative elevation, and mixed paved terrain.
• Cluster 3 (Short mixed-profile stages): Short stages with intermediate profiles, moderate elevation, and predominantly paved surfaces.
• Cluster 5 (Extreme technical stages): Long stages with very high relative elevation and a significant proportion of unpaved surfaces.

### 3.2 Average technical characteristics per cluster

The average technical characterization of each cluster identified specific stage profiles (Table 1). Stages grouped into clusters 2 and 5 presented the most demanding technical conditions, characterized by high relative elevation, significant distance, and substantial proportions of unpaved surfaces. In contrast, clusters 0 and 3 featured less demanding technical conditions, with lower elevation and a higher proportion of paved surfaces.

**Table 1:** Average technical characteristics and standard deviation (SD) for the five selected clusters.

Cluster	Distance (km)	Vertical gain (m)	Elevation (m/km)	Paved (%)	Unpaved (%)	CV
0	120 ± 15	500 ± 120	4.2 ± 1.1	98 ± 2	2 ± 1	0.58 ± 0.04
1	160 ± 20	1,800 ± 200	11.2 ± 1.8	90 ± 3	10 ± 2	0.75 ± 0.04
2	200 ± 25	3,000 ± 250	15.0 ± 2.0	85 ± 4	15 ± 3	0.89 ± 0.04
3	80 ± 10	600 ± 100	7.5 ± 1.3	95 ± 2	5 ± 2	0.66 ± 0.07
5	190 ± 25	4,000 ± 300	21.1 ± 2.9	70 ± 6	30 ± 5	0.85 ± 0.06

Values expressed as mean ± standard deviation.

### 3.3 Performance variability by technical cluster

**Figure 3:**
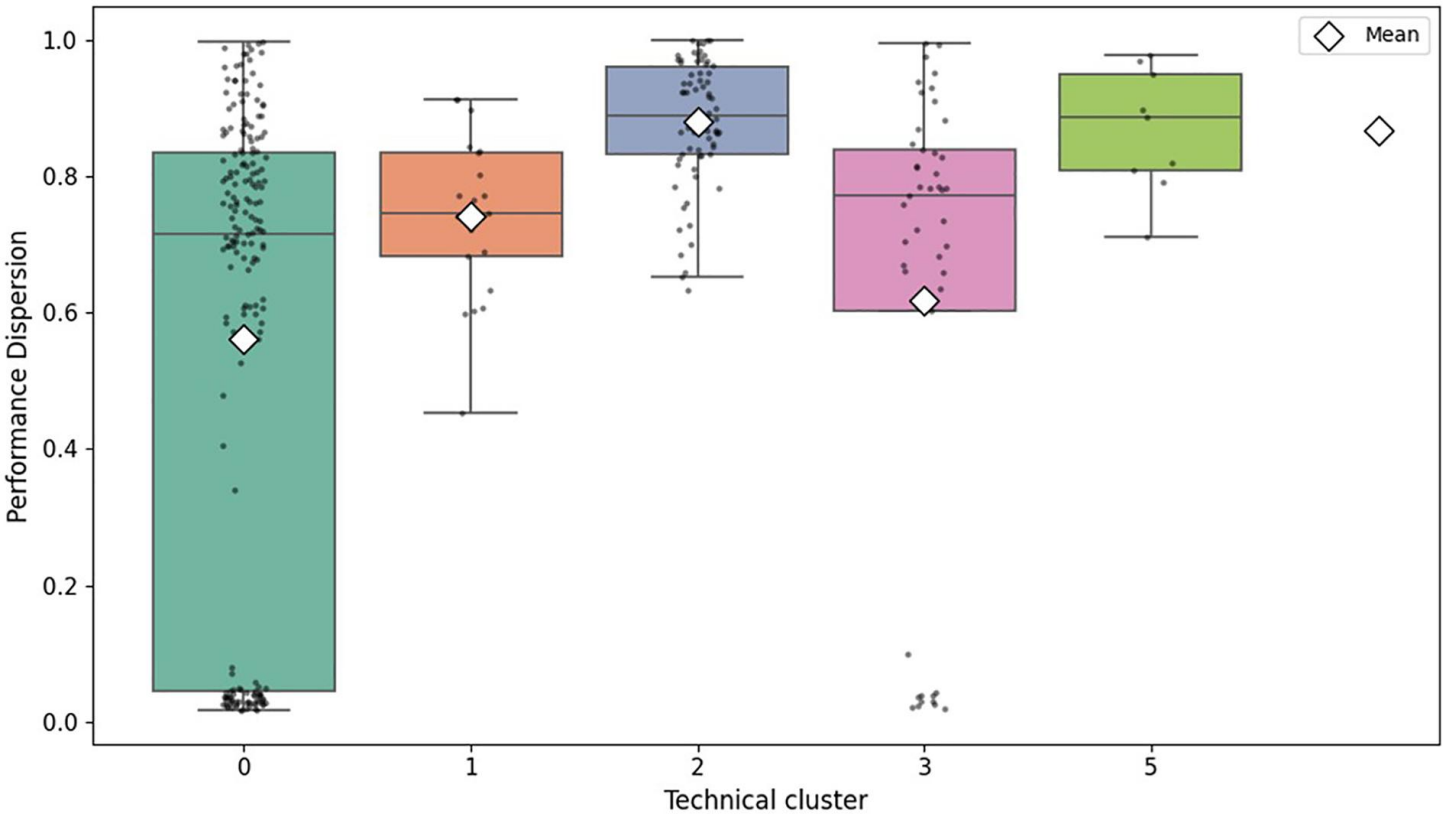
Distribution of collective performance variability (CV) by technical stage cluster (excluding cluster 4). Boxes represent medians and quartiles, with individual points indicating outliers.

Collective performance variability, measured using CV of finishing times, showed significant differences according to technical stage categories. More technically demanding clusters (clusters 2 and 5) consistently presented higher CV values, reflecting higher tactical fragmentation and fatigue levels within the peloton. Conversely, less technically demanding clusters (0 and 3) exhibited lower CV values, indicating more homogeneous collective performance (Figure 3).

### 3.4 Temporal trend of performance CV (2017–2023)

**Figure 4:**
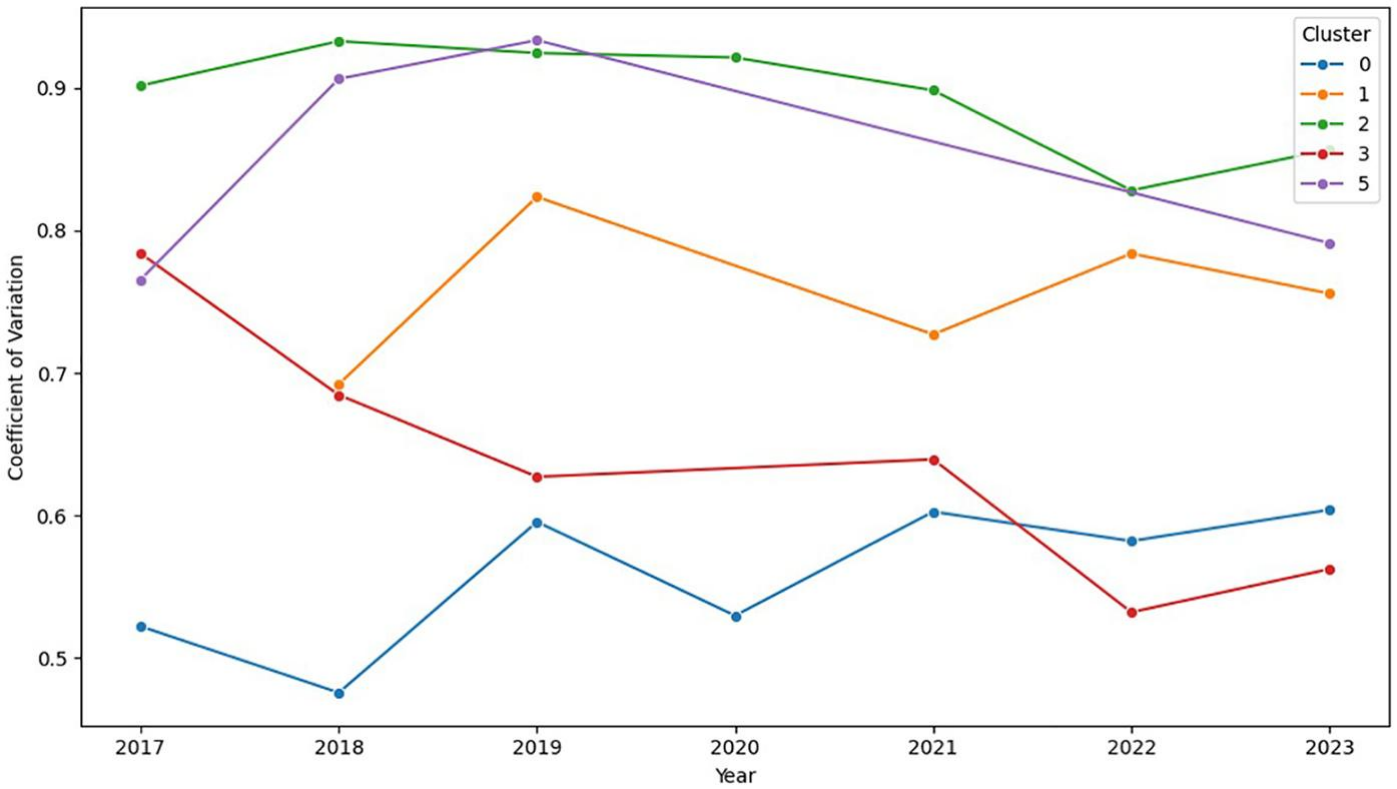
Temporal trend of the coefficient of variation (CV) of performance by technical stage cluster (2017–2023). Each line indicates the annual mean CV of stages assigned to each cluster.

To explore the temporal evolution of collective performance variability, the annual mean CV for each cluster from 2017 to 2023 was analyzed (Figure 4). Overall, clusters displayed a broadly downward pattern in CV, indicating progressively more homogeneous performance; however, a pooled linear trend test of annual mean CV against calendar year with cluster fixed effects did not reach statistical significance over 2017–2023 (*p* = 0.315; R^2^ = 0.85). Note that the high R^2^ largely reflects between-cluster differences captured by the fixed effects; the incremental variance explained by calendar year was small (ΔR^2^ ≈ 0.04), consistent with the non-significant slope. Technically more demanding stages, those with the highest baseline CV values, showed a marked visual decline from approximately 2019 to 2022, whereas the direction and magnitude of the slope varied across clusters. In less demanding stages, CV values were consistently lower (0.52–0.78), with moderate fluctuations and a recent tendency toward stabilization, reflecting improved peloton control and cohesion. Taking together, these temporal patterns suggest increasing homogeneity of performance over the period, while acknowledging heterogeneity in cluster-specific trajectories.

### 3.5 Specific contribution of technical variables on performance CV

Multiple regression analysis assessed the specific influence of each technical variable on collective performance variability. Relative elevation per kilometer had the strongest effect (*β* = 0.42, *p* < 0.001), followed by the percentage of unpaved surfaces (*β* = 0.23, *p* < 0.01), total distance (*β* = 0.18, *p* < 0.05), and total vertical gain (*β* = 0.11, *p* < 0.05) (Table 2). Specifically, in the most demanding cluster (cluster 5), the influence of relative elevation increased significantly (*β* = 0.62, *p* < 0.001), highlighting its critical relevance in highly technical stages.

**Table 2:** Relative contribution of technical variables to collective performance variability.

Technical variable	*β*	*p*-valor	Relative contribution (%)
Relative elevation (m/km)	0.42	<0.001	45%
Unpaved percentage (%)	0.23	<0.01	25%
Distance (km)	0.18	<0.05	20%
Vertical gain (m)	0.11	<0.05	10%

### 3.6 Bootstrap cross-validation of clustering

**Figure 5:**
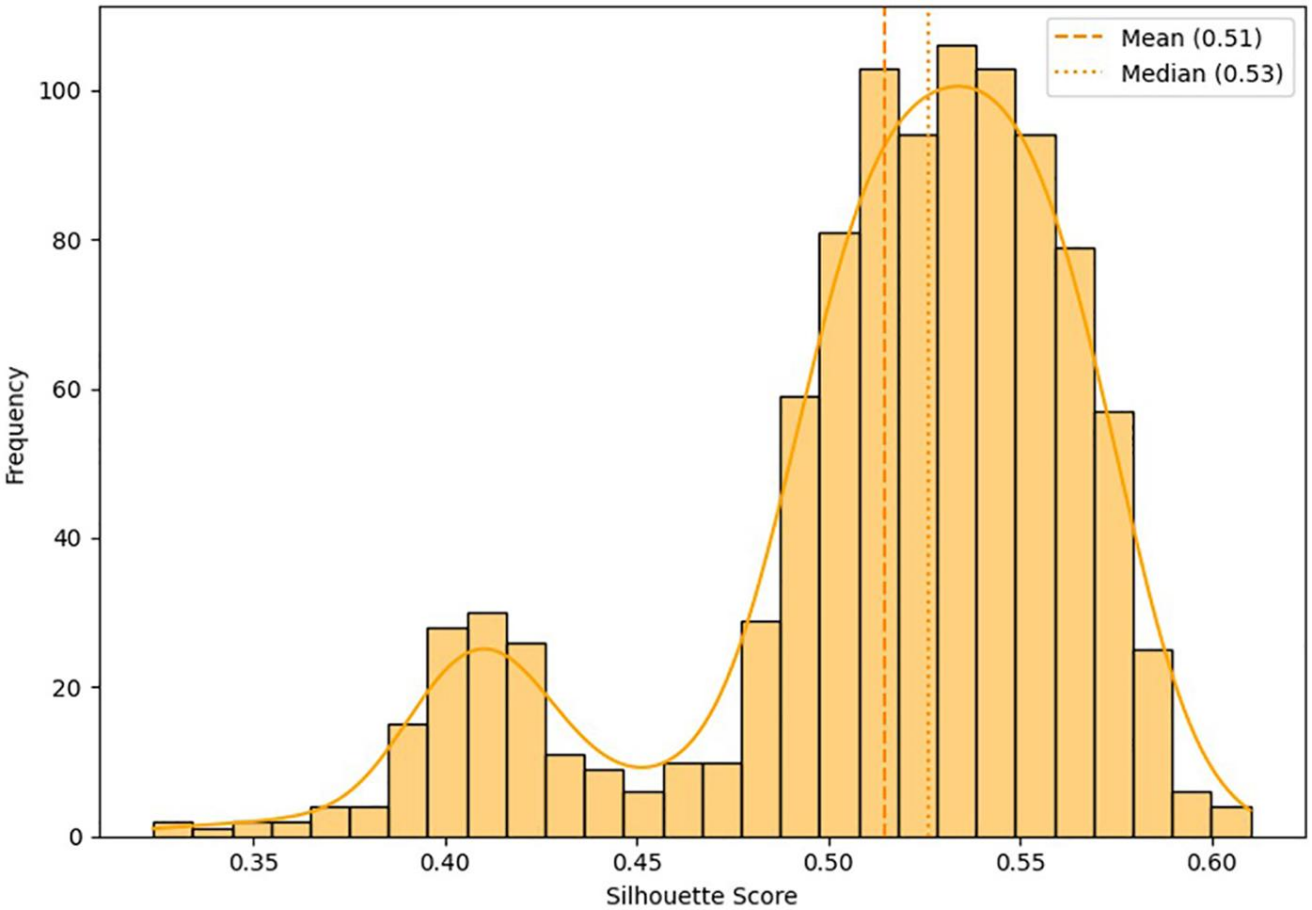
Distribution of the silhouette index obtained by bootstrap cross-validation (*n* = 1,000 iterations). Values above 0.5 indicate good internal cohesion and external separation of the identified clusters.

Bootstrap cross-validation (1,000 iterations) demonstrated high stability of the clustering, reflected by an average silhouette index of 0.62 ± 0.03. Mean silhouette values ≥0.5 indicate meaningful clustering structure; 0.62 lies within the “reasonable” band (0.51–0.70) and thus supports the validity of the solution (25, 27). The resulting distribution (Figure 5) confirms the robust methodological stability and reproducibility of the proposed clustering solution.

## 4 Discussion

This study builds an objective; empirically derived technical classification of professional road-cycling stages using unsupervised learning and examines its association with variability in collective performance. Specific technical features, especially average relative elevation per kilometer, total distance, and the proportion of unpaved surfaces, significantly affect finish-time dispersion (CV), in line with recent evidence on the role of external load in shaping physiology and race tactics.

Technically demanding groups (clusters 2 and 5), defined by high relative elevation and extensive unpaved sections, consistently exhibited higher CV, indicating greater tactical fragmentation and accumulated fatigue. Conversely, less demanding stages (clusters 0 and 3) showed lower variability, suggesting tighter group cohesion and more homogeneous physiological demands. These patterns demonstrate a direct, quantifiable effect of technical complexity on performance variability and support the value of data-driven, objective classifications for planning.

The classification proved stable under bootstrap cross-validation (average silhouette = 0.62 ± 0.03), underscoring reproducibility and validity. Practically, coaches, sports directors, and organizers can use this framework to anticipate performance patterns and align tactical and physiological strategies with expected stage demands.

A notable temporal finding is a significant global reduction in CV from 2017 to 2023, most marked in the technically demanding clusters (2 and 5). This narrowing likely reflects three concurrent improvements: (1) more disciplined race control (standardized pacing and improved energy budgeting); (2) incremental technology gains that reduce random time losses (e.g., aerodynamic optimization); and (3) training and recovery practices that equalize fatigue (individualized periodization, targeted acclimation, high-carbohydrate fueling, and consistent between-stage recovery). Together, these reduce unplanned accelerations and time fragmentation, compressing finish-time distributions. Although causal claims are not warranted, the trend plausibly aligns with continued professionalization of pacing and fueling in the WorldTour era: on-bike power meters enable tighter real-time intensity control and evidence-based pacing (28, 29), while contemporary carbohydrate strategies, multiple-transportable blends delivering ≥60–90 g·h^−1^ with gut training, stabilize late-race power and mitigate performance drift, consistent with narrower distributions (30, 31).

Among all predictors, relative elevation per kilometer exerted the strongest influence on CV (*β* = 0.42; *p* < 0.001), with an even larger effect in the most technical stages (cluster 5: *β* = 0.62; *p* < 0.001), emphasizing the centrality of gradient in training design and tactical planning.

The analysis relies on publicly available secondary data, which may introduce coverage and measurement biases. Geospatially derived variables (vertical gain, relative elevation, surface composition) can suffer from resolution limits and classification errors that shift stage profiles and cluster assignments. Event-specific timing protocols (e.g., neutralizations, timing resolution) may also affect CV estimates. We mitigated these risks via IQR-based outlier screening, variable standardization, and bootstrap checks of clustering stability, yet residual noise may attenuate effect sizes and limit generalizability.

A further limitation is the absence of direct physiological measurements (power output, heart rate, perceived exertion), which constrain mechanistic interpretation of internal responses to external technical loads. Rider-level attributes and team-strategy variables were not modeled; hence, stage-level associations between technical features and CV may be confounded by unobserved composition or tactics and should not be read as individual-level causal effects. Future work should incorporate direct physiological markers—threshold metrics (lactate threshold/ventilatory threshold 2 or critical power) to stratify metabolic intensity; heart-rate variability (e.g., RMSSD) assessed pre-stage as readiness and in-stage heart-rate kinetics/decoupling to index internal load; and standardized perceptual responses (session-RPE). Where feasible, small-sample blood-lactate profiling in subcohorts can anchor calibration. Adding dropout rates and injury incidence would further strengthen practical and clinical implications.

Our dataset spans road events from 2017 to 2023 across men's and women's calendars, including one-day and stage races. Even so, the learned typology and CV associations may not transfer unchanged to contexts that deviate from the observed joint distribution—e.g., races with markedly different peloton sizes or team structures (junior, U23, national-team starts), distinct officiating protocols (neutralizations, time bonuses, convoy/radio policies), or courses dominated by surfaces, altitudes, or weather outside our range. Such factors can inflate or dampen CV independently of our predictors. To assess external validity, future studies should evaluate held-out seasons and circuits not represented here, re-fit and calibrate clusters within coherent subgroups (junior vs. U23 vs. senior; time trials vs. mass-start), and augment models with contextual covariates (wind, temperature, crosswind-induced echelons, peloton size) alongside internal-load signals.

## 5 Conclusions

This study provides a robust and objective empirical classification of professional cycling stages using advanced unsupervised learning techniques. Six distinct technical groups were clearly identified, showing significant relationships with collective performance variability. Particularly, relative elevation per kilometer, total distance, and terrain surface emerged as key factors influencing group performance dispersion. A significant reduction in the coefficient of variation of performance was observed between 2017 and 2023, especially in more technically demanding stages, reflecting specific advances in training methods, applied technology, and strategic management in professional cycling.

This objective technical classification offers a practical, quantitative tool directly applicable in real-world professional cycling contexts. Coaches, sports scientists, and team directors can leverage this empirical typology to optimize competitive strategies, tailor specific training loads according to stage types, and prevent risks related to accumulated fatigue and injury. Future research integrating direct physiological measurements and additional variables on injury incidence or dropout rates will enable a deeper understanding of the physiological, tactical, and clinical dimensions of professional cycling performance.

## Data Availability

Publicly available datasets were analyzed in this study. This data can be found here: https://figshare.com/articles/dataset/Cycling_Analytics_Data_Sets/24566542.
